# MicroRNAs as clinical biomarkers?

**DOI:** 10.3389/fgene.2015.00240

**Published:** 2015-07-14

**Authors:** Timothy G. Angelini, Costanza Emanueli

**Affiliations:** ^1^Royal Surrey County HospitalGuildford, UK; ^2^Bristol Heart Institute, School of Clinical Sciences, University of BristolBristol, UK; ^3^National Heart and Lung Institute, Imperial College LondonLondon, UK

**Keywords:** microRNAs, clinical biomarkers, exosomes, cardiac disease, myocardial infarction

A clinical biomarker has been defined as “any cellular, biochemical, molecular, or genetic alterations by which a normal, abnormal, or simple biological process can be recognized or monitored” (Drucker and Krapfenbauer, 2003; Rahim et al., 2015). Biomarkers are widely employed throughout medicine on a day to day basis to diagnose, prognosticate, and predict outcomes of disease and illness or even as guides of treatment. They can either be used as a standalone, or more commonly, in conjunction with other test results. Moreover, biomarkers are often chosen as “surrogate endpoints” in clinical trials. Biomarkers must fulfill certain criteria in order to make it in to regular use in clinical practice. They must be specific to the condition that they are indicated in, sensitive, and must be practical in terms of accessibility of the sample, ease of testing method and provide information in which to guide clinical decisions. However, many biomarkers have unsatisfactory specificity or are not as sensitive as they are made out to be. In addition, biomarkers for some conditions are not yet available. Consequently, research in to novel biomarkers should be encouraged.

Biomarkers can be classified in to different classes, one being the sources where they measured from. In this respect, there are two distinct kinds of biomarker; intracellular, and extracellular (which can be broken down into three further groups by means of sampling; invasive, minimally invasive, and non-invasive). Examples of intracellular biomarkers include many of those used in oncology, such as BRCA1/2 (Drooger et al., 2015), and estrogen receptor and hormone receptor status in breast cancers (Yang et al., 2014), and BRAF genes in melanoma (Dar et al., 2015). For these tests, the cells from the suspected tumor must be invasively sampled, often by core sampling, or tissue scrapings. Extracellular biomarkers are often but not always linked with less invasive sampling, for example the withdrawal of a peripheral venous blood sample or urine collection. Examples are urine test for beta-human chorionic gonadotrophin (Chen et al., 2015) as an indicator of pregnancy or prostate specific antigen (PSA) (Ravery, 1999) in a suspected prostate cancer case.

An exciting, but not yet conclusive, new area of research with the potential to derive new clinical biomarkers has been into microRNAs (miRs). These short RNA molecules were discovered in 1993 (Lee et al., 1993) in *C. elegans* and found present in human samples in 2000 (van Rooij, 2011). From there, their love story with the biomedical community has seen a crescendo of enthusiasm and they have been highly regarded as both novel therapeutic targets, and possible intracellular and extracellular biomarkers. We might speculate that the reason behind this popularity is two-folds:

1. The mode of action of miRs is relatively straightforward. MiRs do not contain any coding for protein production (and hence they are classified as noncoding RNAs–ncRNAs) and each miR is capable of post-translational regulation of the expression of a plethora of target messenger RNAs (mRNAs), which it recognizes through the semi complementary nucleotide base-pairing between its “seed sequence” (of just eight nucleotides) and one or more miR binding sites in the 3′-untranslated region of the mRNA targeted (Jackson and Standart, 2007). The biology of miR appears much simpler in comparison to, as an example, the long ncRNA that act via multiple, with still largely un-clarified mechanisms, at transcriptional and post-transcriptional levels, in the nucleus and the cytosol, and in *cis* and *trans* (Rinn and Chang, 2012). Moreover, miRs exist in a limited number, thought to be around 2000 in humans. However, miRs variants are still being discovered through RNA-sequencing approaches.
2. MiR are released by their producing cells in protected forms allowing them to remain for long periods in biological fluids. Moreover, miRs were initially believed to be tissue- and even cell-specific. This led to the assumption that what was found in the blood could sense the altered status of the cell types or tissues that were supposed to produce such miR. Now the concept of tissue/cell-specificity has been largely dismissed and it is becoming clear that at best we can talk of cell/tissue type enrichment for most, if not all, the known miRs. Hence, it is quite unrealistic to think that, under most clinical scenarios (with exceptions discussed later), the level of a single miR measured in the whole plasma or serum can be informative of a local slowly or relatively slowly evolving condition, such as, a developing cancer (Washam et al., 2013).

Circulating individual miRs, such as miR-21 (suggested to be useful for the detection of various carcinomas) (Wang et al., 2014; Wu et al., 2015), and circulating groups of miRs (such as a serum miR classifier encompassing miR-29a, miR-29c, miR-133a, miR-143, miR-145, miR-192, and miR-505, that has been proposed to detect hepatocellular carcinoma) (Lin et al., 2015) have been suggested as potential biomarkers that could be used in cancer detection and staging, and to follow-up already diagnosed cancer patients. However, in a recent article reporting the results of the comparison among 15 previous reports on potential new breast cancer biomarkers (Leinder et al., 2013) there was a scarce overlap between results: “Of the 143 circulating miRNAs reported to be differently regulated, 100 were supported by just 1 reference; 25 others had discordant results across publications and of the remaining 18 miRs, 8 had fold changes too low to be confirmed. Of the 10 concordant results, 9 were supported entirely by publications from the same institution and had authors in common” (Leinder et al., 2013; Witwer, 2015). This suggests that further efforts are needed before miR-based biomarkers can benefit cancer patients and that this could also apply to other disease conditions. Cancer has been the first translational area for miR work, closely followed by heart failure. Looking at both clinical scenarios, we find a typical example of unspecificity: circulating miR-21 has been proposed as a biomarker of both prostate cancer (Egidi et al., 2013) and myocardial fibrosis (in heart failure) (Thum et al., 2008). miR-21 is also the most expressed miR by vascular endothelial cells (Greco and Martelli, 2014), which are the cells directly lining the circulating blood and for this reason supposed to be the highest contributors to miRs circulating in the peripheral blood (Greco and Martelli, 2014; Witwer, 2015). A miR such as this, amongst others that are widely and highly expressed, might not be an ideal circulating candidate biomarker, suggesting that miRs that are usually under expressed, but upregulated under a particular condition could be better suited to be employed in a diagnostic test. For example, we recently found that miR-503 appears in the blood of diabetic patients at the last stage of critical limb ischemia, i.e., when they need an amputation (Caporali et al., 2011). It is possible that circulating miR-503 could have a diagnostic/prognostic value when measured at the earlier stages of the disease, however, measuring miR-503 in a small leg muscle biopsy could provide more reliable information.

There are areas where we believe that circulating miRs show more promise and this is in the recognition of acute events, such as myocardial infarct (MI), as well as in the surgical setting, where time-restricted changes in miR expression have been reported consistently. For examples, the heart (and skeletal muscle)—enriched miR-1 has been noted to increase in patients after open heart surgery, after MI or transcoronary ablation of septal hypertrophy, an interventional procedure that mimics MI (D'Alessandra et al., 2010; Widera et al., 2011; Liebetrau et al., 2013; Nabialek et al., 2013). Diagnostic biomarkers are a key part in emergency service provision and the rapid diagnosis, and therefore treatment of patients with life threatening conditions. One of the most widely used in the emergency department are cardiac troponins (cTns: cTn-T and cTn-I) for the diagnosis of MI. CTns are used in conjunction with other investigations, such as electrocardiogram (ECG) changes, allowing, for example, to determine whether a patient has had a “STEMI” type MI (with ST segment Elevation by ECG). ECG cannot pick up non-STEMI cases and here laboratory biomarkers are highly important (Alpert et al., 2000). However, cTns are not always specific to MI, they can be raised in patients with other cardiac conditions and also after infection. In light of this, research into miRs as potentially better biomarkers has been carried out showing time-dependent increases in cardiac-enriched, ischemia-responsive miRs in the blood of MI patients (D'Alessandra et al., 2010; Nabialek et al., 2013). There has also been claim that miRs can help differentiating the diagnosis of a STEMI compared with other myocardial conditions, such as stable angina, non-STEMI, and Takotsubo cardiomyopathy (Nabialek et al., 2013; Ward et al., 2013; Jaguszewski et al., 2014). However, it is yet to be demonstrated that miRs can replace cTns as routine biomarkers used in the intensive coronary care unit, in depth investigations of specificity and sensitivity in different cohorts or patients are needed. Additionally, in the realm of emergency medicine, sensitivity and specificity of miRs response in terms of circulating changes are not the only issues, because the time necessary by this putative biomarker to appear elevated in the blood or another biological fluid is critical. Classically, cTns take a few hours to increase in the blood after an MI. MiR-1 has been proposed to go up earlier than cTns (Liebetrau et al., 2013). However, for these comparisons, the time to obtain the test results and the test reproducibility are big obstacles yet to be overcome. Differently from high sensitive cTns that today are measured by immune-enzymatic reactions allowing for results to be obtained in around 20 min, PCR-based miR analyses are still quite time-consuming. Alternative techniques for miR quantification have been proposed (Arata et al., 2012) but they are far away from being commonly employed by the scientific community, let alone the clinical diagnostic lab. Additionally, in a clinical laboratory staff cannot reason as in observational studies, where everybody is often quite satisfied with saying “miR-1 is increased in the blood of MI patients in comparisons to a control group.” Diagnostic use of miRs needs a different rigor and first of all a definition of what the “normal” threshold of miR concentration is, above which we can suspect in a patient who is experiencing a heart attack. Scientists working in the miR field are aware that this is not an easy task and by using PCR-based methods quantitative differences between different studies are common. In addition, the data normalization approaches are still debated and interference by heparin (used in interventional procedures) with the PCR reaction has been reported (Mayr et al., 2013), even if protocol to nullify the “heparin effect” are adopted. Alternative technologies can be developed, but this will require further investment, time and validation efforts (Arata et al., 2012).

In conclusion, We believe that miRs hold potential value as clinical biomarkers, but, their journey to the diagnostic lab is still long and needs improved approaches at multiple levels, starting with technical refinement in the miR concentration evaluation, the use of RNA-sequencing to possibly recognize new miRs that are better candidates (higher tissue/cell-specificity, lower expression under healthy conditions etc.) and the use of blood fractions potentially enriched in miRs (like exosomes and microparticles) for diagnostic tests. It is also possible that miR clusters have more specificity than single miRs. Moreover, miR could be associated to other biomarkers to improve the diagnostic power.

## Funding and acknowledgments

This work was funded by the National Institute of Health Research (NIHR) Bristol Cardiovascular Biomedical Research Unit (BRU) The views expressed are those of the Authors and not necessarily those of the NHS, the NIHR or the Department of Health. CE is a PI in the Leducq transatlantic network in vascular microRNAs (MIRVAD).

### Conflict of interest statement

The authors declare that the research was conducted in the absence of any commercial or financial relationships that could be construed as a potential conflict of interest.
